# Improving Social Communication With TimeSlips Among Persons Living With Dementia: Results From a Pilot Study

**DOI:** 10.1093/geroni/igaa057.3060

**Published:** 2020-12-16

**Authors:** Ranjini Mohan, Kyong Hee Chee, Seoyoun Kim, Olga Gerhart

**Affiliations:** 1 Texas State University, Round Rock, Texas, United States; 2 Texas State University, San Marcos, Texas, United States

## Abstract

Persons living with dementia (PLWD) often display declining communication and conversational skills, leading to reduced quality of life and social isolation. Given limited evidence for interventions targeting conversation skills in PLWD, we examined the transcripts of a creative group storytelling program, TimeSlips, offered in a memory care community in Central Texas. We analyzed communicative exchanges using modified rules of the ‘Conversational Act Profile’ (Fey, 1986). Utterances were coded for each participant’s interaction 1) with the facilitator and 2) with other participants. Preliminary evidence for six participants across six sessions is reported here. While the number and type of participant-to-facilitator utterances did not show a specific pattern, five of the six participants showed increased participant-to-participant communication across sessions. These findings suggest that a creative storytelling program such as TimeSlips may promote greater communication among PLWD, thereby improving social connectedness amongst them and possibly, their quality of life.

